# Discrete element modelling of under sleeper pads using a box test

**DOI:** 10.1007/s10035-018-0795-0

**Published:** 2018-03-17

**Authors:** Huiqi Li, Glenn R. McDowell

**Affiliations:** Nottingham Centre for Geomechanics, University of Nottingham, Nottingham, UK

**Keywords:** DEM, Railway ballast, Under sleeper pad

## Abstract

It has recently been reported that under sleeper pads (USPs) could improve ballasted rail track by decreasing the sleeper settlement and reducing particle breakage. In order to find out what happens at the particle–pad interface, discrete element modelling (DEM) is used to provide micro mechanical insight. The same positive effects of USP are found in the DEM simulations. The evidence provided by DEM shows that application of a USP allows more particles to be in contact with the pad, and causes these particles to transfer a larger lateral load to the adjacent ballast but a smaller vertical load beneath the sleeper. This could be used to explain why the USP helps to reduce the track settlement. In terms of particle breakage, it is found that most breakage occurs at the particle–sleeper interface and along the main contact force chains between particles under the sleeper. The use of USPs could effectively reduce particle abrasion that occurs in both of these regions.

## Introduction

**Fig. 1:**
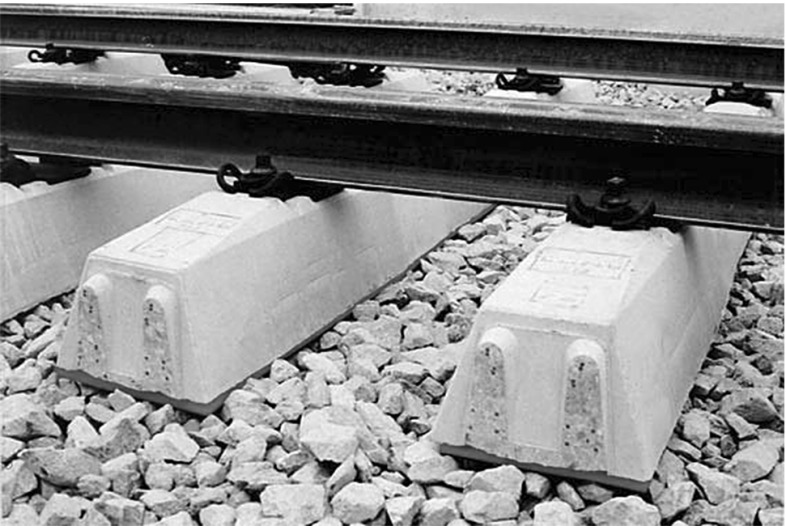
Ballasted track with under sleeper pads

In recent years, under sleeper pads (USPs) have become popular in newly-built high speed railway tracks in central Europe. USPs are resilient pads installed at the bottom surfaces of sleepers to provide an intermediate elastic layer between the ballast and the sleeper with the intention of improving sleeper–ballast interaction or for mitigating ground borne noise and vibration. Figure 1 shows a picture of a typical ballasted railway track with USPs. USPs normally have a thickness of about 10 mm and are made of polyurethane elastomer with a foam structure including encapsulated air voids [1].

A number of field and laboratory tests have been carried out to investigate the influence of USPs, mostly reporting positive results, a summary of which will now be given. From field experience, Bolmsvik [2] showed that track misalignment could be reduced by the use of USPs. USPs have also been found to reduce both inter-particle abrasion and sleeper–ballast attrition [3, 4]. Both Riessberger [5] and Abadi et al. [6] observed that the use of a USP increases the ballast–sleeper contact area which leads to a reduced contact pressure in experimental tests. This was presumed to be the reason why USPs help to reduce ballast damage [3]. Lakušić et al. [7] reported both the lateral stability and load distribution from the sleepers were improved due to ballast embedding into USPs. Baghsorki et al [8] found that USPs also reduced the sleeper settlement in their lab tests, although no physical explanation was given. Loy [9] simply claimed that using USPs ‘the track superstructure will always exhibit more favourable characteristics than a structure without USPs’. Although many advantages of USPs have been identified from field experience or laboratory tests, none have provided comprehensive evidence to show exactly how USPs work. A better understanding of the influence of USPs on sleeper–ballast interaction is therefore needed.

Discrete element modelling (DEM) pioneered by Cundall and Strack [10] provides a powerful tool to investigate granular material at a micro level. In the past decade, DEM has been successfully applied to railway ballast. The early studies of DEM modelling ballast particles [11, 12] represented a particle as a simple sphere with the aim of investigating ballast degradation by using the particle replacement method. Lu and McDowell [13] showed that the use of irregular particle shape in DEM could provide more particle interlock and thus gave much reduced particle rotations and displacements. A few methods to generate complex shapes of ballast particle were later proposed [14–17]. With the use of irregular shapes, the shear strength of ballast particles has been successfully modelled by triaxial simulations [18–21]. The improvement of using geogrid-reinforced ballast has also been successfully modelled [22–25] by representing the geogrid as a group of bonded spheres, which is similar to the generation method for the USP in this study. In this paper, DEM is employed to simulate the behaviour of an under sleeper pad in a box test developed by McDowell [26]. The USP is modelled by three layers of hexagonal-closed-packed, bonded spheres. The simulation results are firstly quantitatively compared with the first 15 cycles of experimental results in Baghsorki et al [8] and then the role of the USP in the sleeper–ballast interaction is studied. The influence of stiffness of the pad on trackbed stiffness and permanent settlement is investigated and particle abrasion is also considered to confirm the effectiveness of the USP in maintaining track quality.

**Fig. 2:**
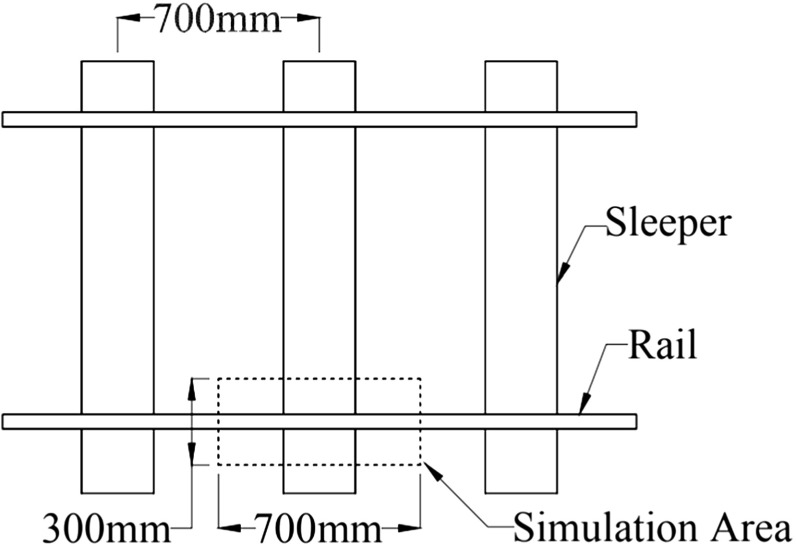
Simulated track area of box test

## Discrete element modelling of a box test

**Fig. 3:**
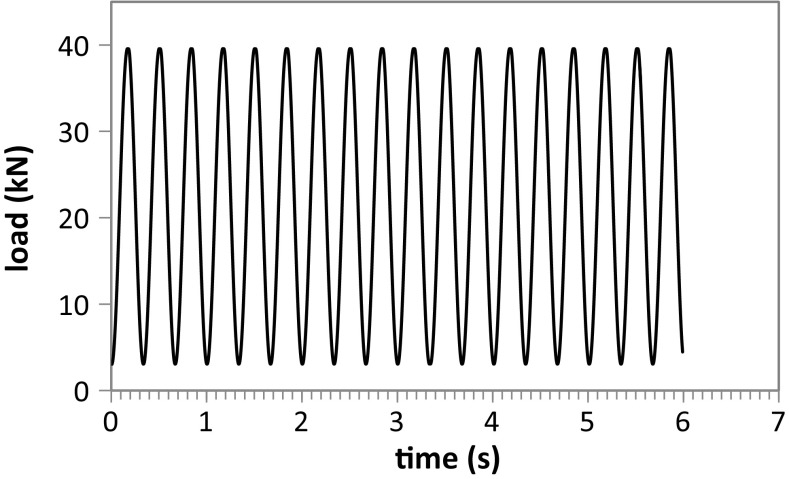
Loading path of the sleeper

The box test which is used to model the ballast–sleeper interaction that occurs under the rail seat of a track (Fig. 2) has been used with success in previous studies [27, 28]. It consists of loading cyclically a section of sleeper (0.3 m $\times$ 0.25 m $\times$ 0.15 m) embedded into ballast and confined in a 0.3 m x 0.7 m x 0.45 m box. The sleeper section is then loaded vertically with a 3 Hz cyclic load oscillating between 3 and 40 kN (Fig. 3); 15 cycles are loaded for all the simulations in this study. It is not possible at this stage to carry out large numbers of cycles due to computational time; in addition the largest changes in measured quantities are always most pertinent in the first few cycles. The stress level is achieved using a servo control mechanism.

**Fig. 4:**
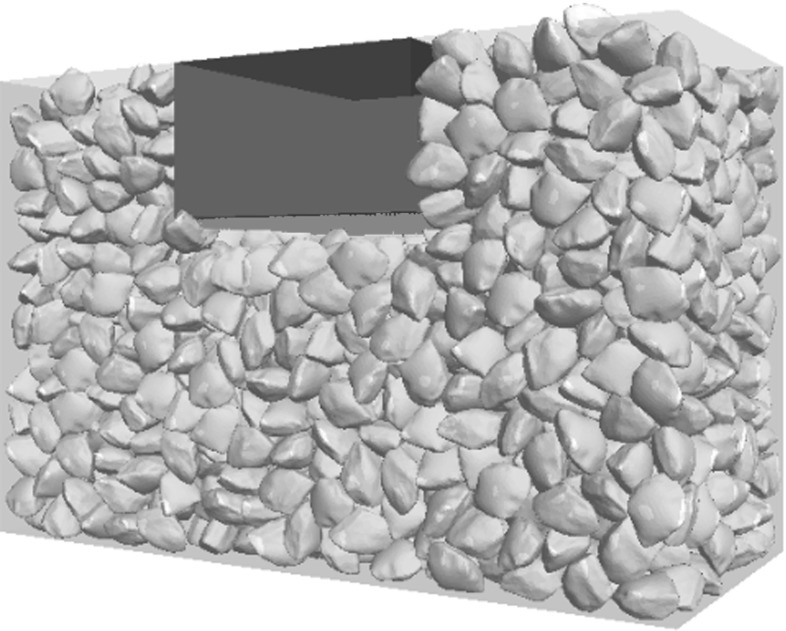
DEM sample of a box test

**Fig. 5:**
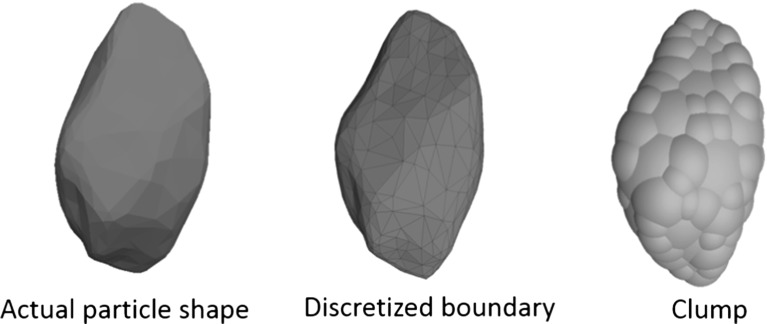
Ballast particle shape

**Table 1:** Input parameters of clump particle and boundary

Properties of clump with real shape	
No. of clumps	1580
Friction	0.5
Possion’s ratio	0.25
Shear modulus	28 GPa
No. of spheres forming clump	41
c_dis	150$^{\circ }$
c_ratio	0.4
Density	2960 $\hbox {kg/m}^{3}$
Damping coefficient	0.7 (default)

Properties of boundary	
Possion’s ratio	0.25
Shear modulus	28 GPa
Friction	0.5

The commercial DEM code PFC3D 5.0 [29] is used in this study. Figure 4 shows the DEM model of the box test. The sample is generated by the following procedure:

1. The ballast particles (modelled using ‘clumps’) are created in a taller box above the box test apparatus and are then allowed to settle under a normal gravity of $-10\hbox { m/s}^{2}$.
2. Change the gravity constant to $-50\hbox { m/s}^{2}$ and then run the sample until equilibrium state to compact the sample.
3. Switch the gravity constant to the normal value of $-10\hbox { m/s}^{2}$ and then run the sample until equilibrium state again. All particles which are not entirely inside the box apparatus or lying inside the boundary of the sleeper are deleted.

The sleeper is simply modelled as a group of elastic walls (Fig. 4). The classic Hertz–Mindlin contact model [29] is used for the ballast particles and they are given a Poisson’s ratio, $\nu = 0.25$ and a shear modulus, $\hbox {G} = 28\hbox { GPa}$ which are typical values for quartz. Lu and McDowell [13] have shown the importance of DEM modelling ballast particle shape in a box test, therefore the ballast particle in this study is modelled by an unbreakable clump [29] with realistic particle shape (Fig. 5). PFC3D is able to create an irregular shaped clump quite simply by using the bubble pack algorithm of Taghavi [30]. The method generally fills a known 3D boundary using spheres of various sizes. It is governed by two parameters: *c_dis* and *c_ratio*. The *c_dis* corresponds to an angular measure of roughness in degrees ( $0<{c}\_{dis}<180$) as defined in Taghavi [30], the *c_ratio* denotes the ratio of smallest to largest sphere forming the clump ($0< c\_{ ratio}<1$). The greater *c_dis* and the smaller *c_ratio*, the smoother the clump surface, they are chosen as 150$^{\circ }$ and 0.4 in this study. The surface of the particle in this study (Fig. 5a) is derived by scanning a real ballast particle using a 3D scanner, as detailed in Li et al [31]. The nodes of the scanned surface are then input to PFC5.0 in the form of STL file. In terms of calculating the inertia tensor, the particle is assumed to have uniform density, the input surface (the actual particle shape in Fig. 5a) is then divided into a set of tetrahedrons formed by the facet vertices and the centre of mass (the discretized boundary in Fig. 5b), the covariance (second moment of mass) of each tetrahedron is then computed and added to an accumulator, at last the covariance matrix is converted to an inertia tensor. This is to say, the inertia tensor of the clump is calculated based on the mesh of input boundary although the forces acting on the clump are calculated based on the spheres forming the clump. In order to avoid non-physical oscillation within the assembly of objects, non-viscous (i.e. inertial) damping reduces their accelerations by 70 % (by default) to allow further dissipation of energy in addition to frictional dissipation at contacts [29]. Table 1 lists all the input parameters of the particles.

**Fig. 6:**
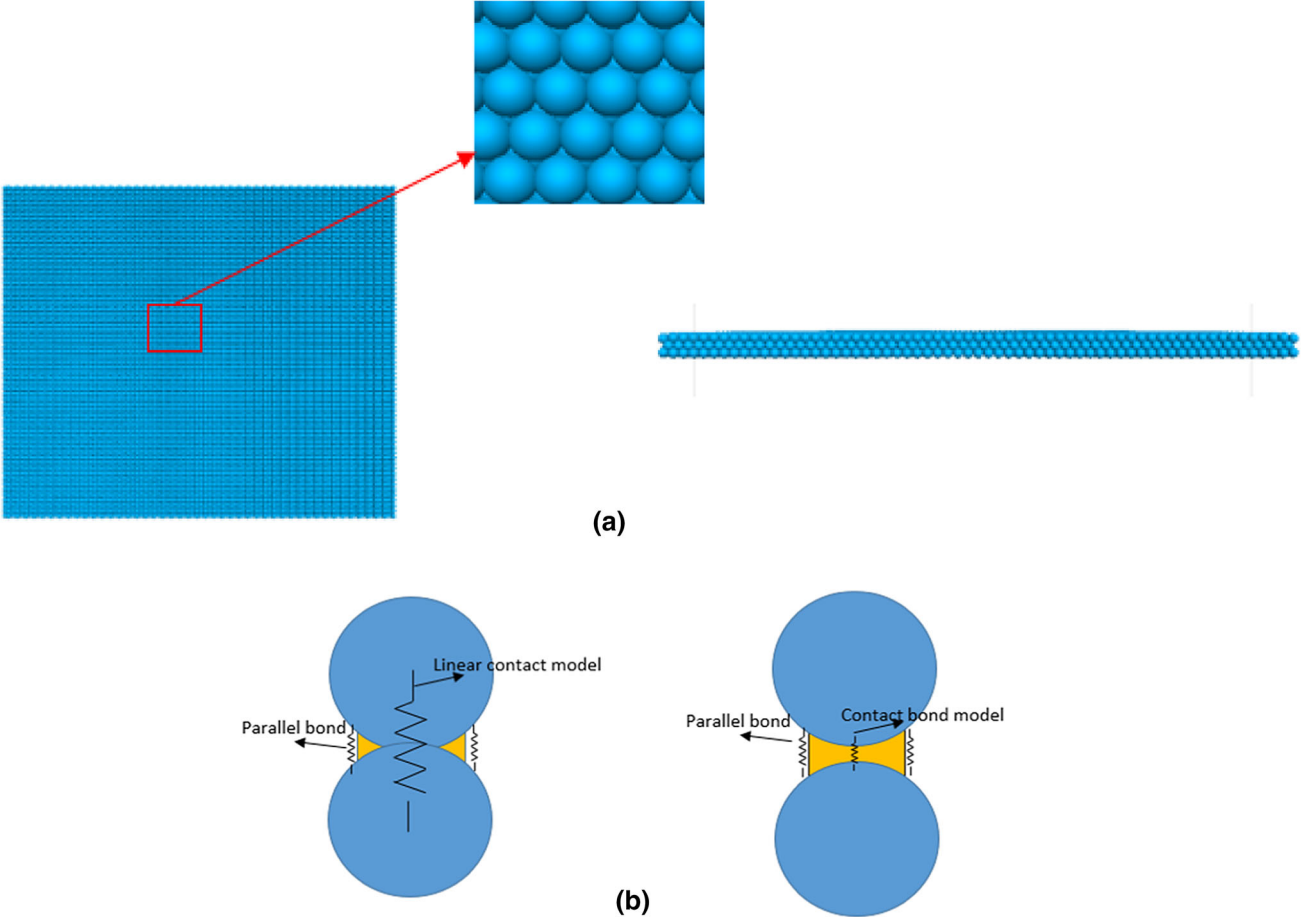
**a** USP used in DEM simulation **b** The ball–ball contact model inside the USP

**Table 2:** The target engineering properties of USP and the characteristic parameters of contact models

Desired engineering properties of USP	
Thickness	9mm
Weight	$5.6\,\hbox {kg/m}^{2}$
Stiffness	0.079–0.105$\hbox { N/mm}^{3}$

Contact models: characteristic parameters	
Linear contact model	Normal stiffness $k_{lcn}$ and Shear stiffness $k_{lcs}$
Contact bond model	Normal stiffness $k_{cbn}$ and Shear stiffness $k_{cbs}$ Normal strength $\sigma _{cbn}$ and shear strength $\sigma _{cbs}$
Parallel bond model	Normal stiffness $k_{pbn}$ and Shear stiffness $k_{pbs}$ Normal strength $\sigma _{pbn}$ and shear strength $\sigma _{pbs}$ and bond radius multiplier $\upalpha$

## Discrete element modelling of under sleeper pad

The USP is formed by 13,550 small spheres with radii of 1.65mm, and are hexagonally-close-packed and bonded together (Fig. 6a). There are three contact models employed at the intra-USP particle contacts: the linear contact model, contact bond model and parallel bond model. The parallel bond acts as a disc of elastic glue at the contact which could transmit both forces and moments so that the USP behaves as a deformable beam. The linear contact model is used for the ball–ball contact to model the resilience of the USP but it is only active when the contact is in compression. In order to give the pad the same stiffness for compression and tension (as Fig. 6b shows), a contact bond model is installed to provide an elastic stiffness working in parallel with the parallel bond when the contact is in tension. The detail principles of contact models are referred to the PFC manual [29] Parallel bonds of the same properties are also installed at the pad–sleeper contacts to make the pad move contiguously with the sleeper during the whole simulation.

**Table 3:** Input parameters of USP

Properties of USP	
No. of mini-spheres	13550
Radius of mini-sphere	1.65 mm
Density	$1400\hbox { kg/m}^{3}$
Damping coefficient	0.7
Friction	0.5
Parallel bond normal and shear stiffness	$4.6\,\times \,10^{7}$ Pa/m
Parallel bond normal and shear strength	1 $\times$ 10$^{60}$ Pa
Parallel bond radius multiplier	1
Contact bond normal and shear stiffness	500.1 N/m
Contact bond normal and shear strength	1 $\times$ 10$^{60}$ N
Normal and shear stiffness of mini sphere	500.1 N/m

Table 2 lists the physical parameters of the USP [8] modelled in this study and also the characteristic parameters for the three contact models The thickness is easily achieved by adjusting the radius of USP spheres and the weight simply depends on the density of mini-sphere. In terms of stiffness, the USP used in the experiment has a value range of $0.079-0.105\hbox { N/mm}^{3}$, so the mean value $0.092\,\hbox {N/mm}^{3}$ is chosen to be the target value in the simulation. As Fig. 6b illustrates, the normal stiffness of the pad in compression $K_{pad\_c}$ can be derived by summing the stiffnesses of the linear contact model and parallel bond model (Eq. 1), whereas the normal stiffness of the pad in tension $K_{pad\_t}$ can be derived by summing the stiffnesses of the contact bond model and parallel bond model (Eq. 2). Considering the unit of target stiffness is in $\hbox {N/mm}^{3}$, the *equations * could be written as:

**Fig. 7:**
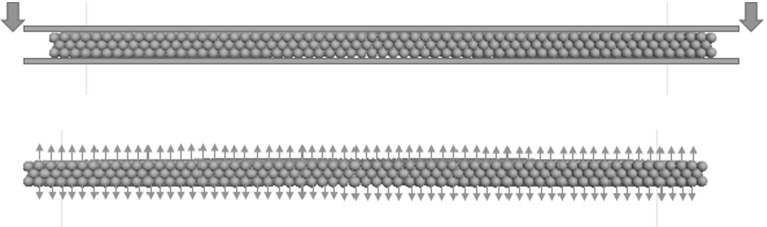
1-d compression test

1 $$\begin{aligned} K_{pad\_c}= & {} \frac{F}{u*A_{ball} }+\frac{F}{u*A_{phb} }=\frac{k_{lcn} }{A_{ball} }+k_{pbn} \end{aligned}$$

2 $$\begin{aligned} K_{pad\_t}= & {} \frac{F}{u*A_{ball} }+\frac{F}{u*A_{phb} }=\frac{k_{cbn} }{A_{ball} }+k_{pbn} \end{aligned}$$

where *F* stands for the compressive force acting on the ball, *u* is the overlap caused by the compression force, $$A_{ball}$$ represents the equivalent loading area of the ball and $$A_{phb}$$ is the area of parallel bond acting at the ball–ball contact. As the balls forming the pad are hexagonal closed packed, the equivalent area $$A_{ball}$$ is assumed to be the area of the square of the same side length as the diameter of sphere. The authors also assume the parallel bond and ball have the same strain under the same compressive force to model a homogenous material. Therefore, by equating the Eqs. 1, 2 to $$0.092\hbox { N/mm}^{3}$$, the normal stiffness of the parallel bond $$k_{pbn}$$ is set to be $$4.6\,\times \,10^{7}$$ Pa/m and the normal stiffnesses of the mini-sphere $$k_{lcn} $$ and the contact bond model $$k_{cbn}$$ are calculated to be 500.1 N/m. The shear stiffness is assumed to be equal the normal stiffness to simplify the calibration process in this study and various values will be investigated in a future study. The shear/normal bond strengths for both contact bond model and parallel bond model are given artificially large values to make the pad unbreakable. The parallel bond radius multiplier $$\alpha $$ [29], which is equal to the parallel bond radius divided by the radius of mini sphere, is assumed to be 1 to give the parallel bond the same diameter with the mini sphere. The input parameters are listed in Table 3.

**Fig. 8:**
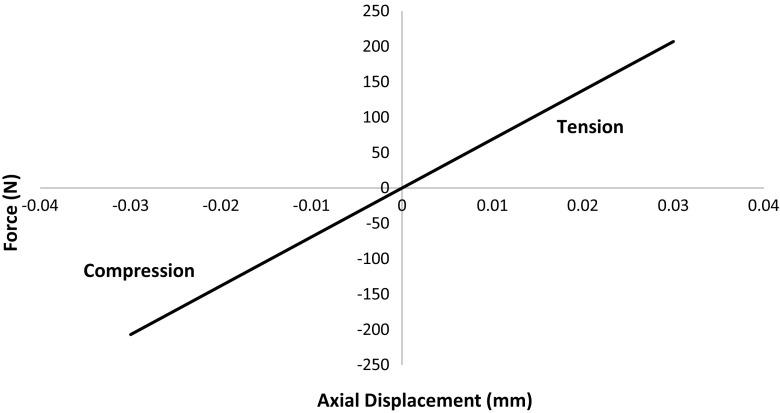
Verification of USP stiffness

These values are then verified by 1D compression/tension test on the pad (Fig. 7). The compression test is simply done by placing the pad between two parallel walls, and then moving the upper wall downwards relative to the fixed bottom wall. The displacement and the resulting force of the upper wall are recorded. For the tension test, a constant velocity was applied at both the upper and lower layers of particles. The axial strain and the total resulting forces at the upper and lower layers of particles are monitored during the test. Figure 8 shows the resulting force as a function of axial displacement in terms of both tension and compression. It shows the stiffness of the pad is exactly $0.092\hbox {N/m}^{3}$. So the USP exhibits linear-elastic behaviour.

**Fig. 9:**
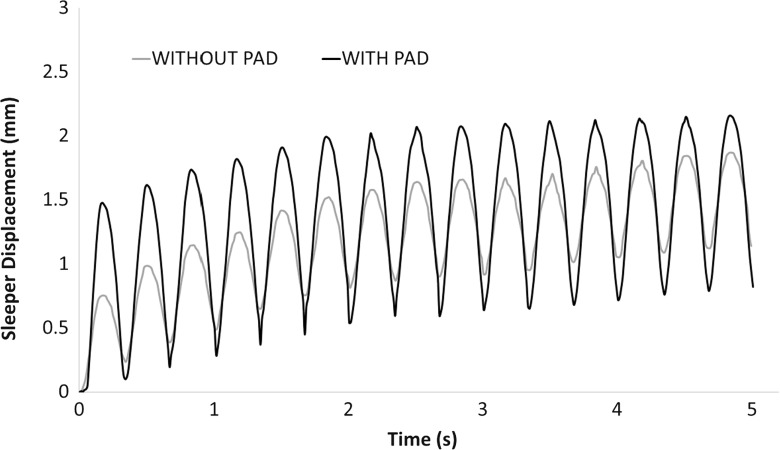
Sleeper deflection versus time for simulations with/without pad

## Results and analysis

Baghsorki et al [8] observed experimentally that the USP reduced the permanent settlement of the sleeper and decreased the trackbed stiffness. The trackbed stiffness is calculated according to Lim [32] as

3 $$\begin{aligned} K=\frac{F_{max} -F_{min} }{\delta _{max} -\delta _{min} } \end{aligned}$$

where *K* denotes trackbed stiffness, $F_{max} -F_{min}$ represents the change in applied loading force on the sleeper and $\delta _{max} -\delta _{min}$ represents the resilient vertical displacement.

**Fig. 10:**
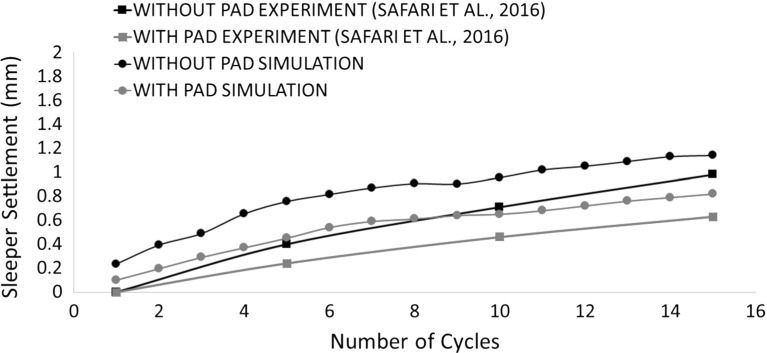
Comparison of DEM with laboratory experiment for sleeper settlement

**Fig. 11:**
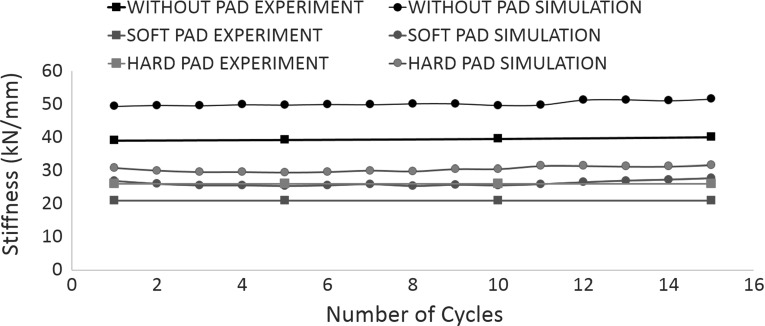
Comparison of DEM with laboratory experiment for trackbed stiffness

Due to the limitation of computation time, the simulations in this study were only repeated twice (i.e., two different random samples) and the average values are used to analysis here. Figure 9 shows the sleeper deflection as a function of time during the whole 15 simulation cycles for both cases with and without the USP. It shows that the pad clearly reduces the permanent settlement and the reduction by using a USP is enlarging with increasing loading time. It is also found that the USP increases the resilient vertical displacement of the sleeper during each cycle, which means a smaller trackbed stiffness corresponding to Eq. 1. Figure 10 records the permanent settlement of each loading cycle (minimum points of each cycle in Fig. 9) and then compares them with experimental results [8]. It can be seen that the permanent settlements predicted by the DEM simulations are only slightly higher than the experimental results. Moreover, the reduction in settlement by applying USP is also estimated. Figure 11 compares the calculated trackbed stiffness of simulations to experimental results. It can be seen that the DEM simulation slightly overestimates the trackbed stiffness for both cases.

**Fig. 12:**
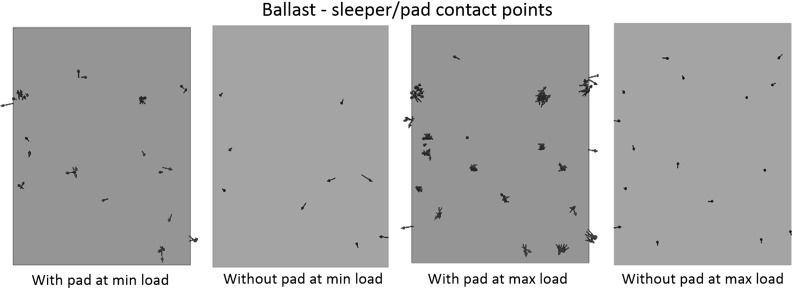
The active contacts at particle–sleeper/pad interface

It is easy to understand why the trackbed stiffness reduces, as the soft pad allows larger resilient settlement during each loading cycle. However it is still necessary to analyse the mechanism as to how the pad effects the permanent settlement. Figure 12 compares the contacts between particle and USP/sleeper at minimum and maximum load during the last loading cycle, which confirms that the USP allows more ballast particles to support the load.

**Fig. 13:**
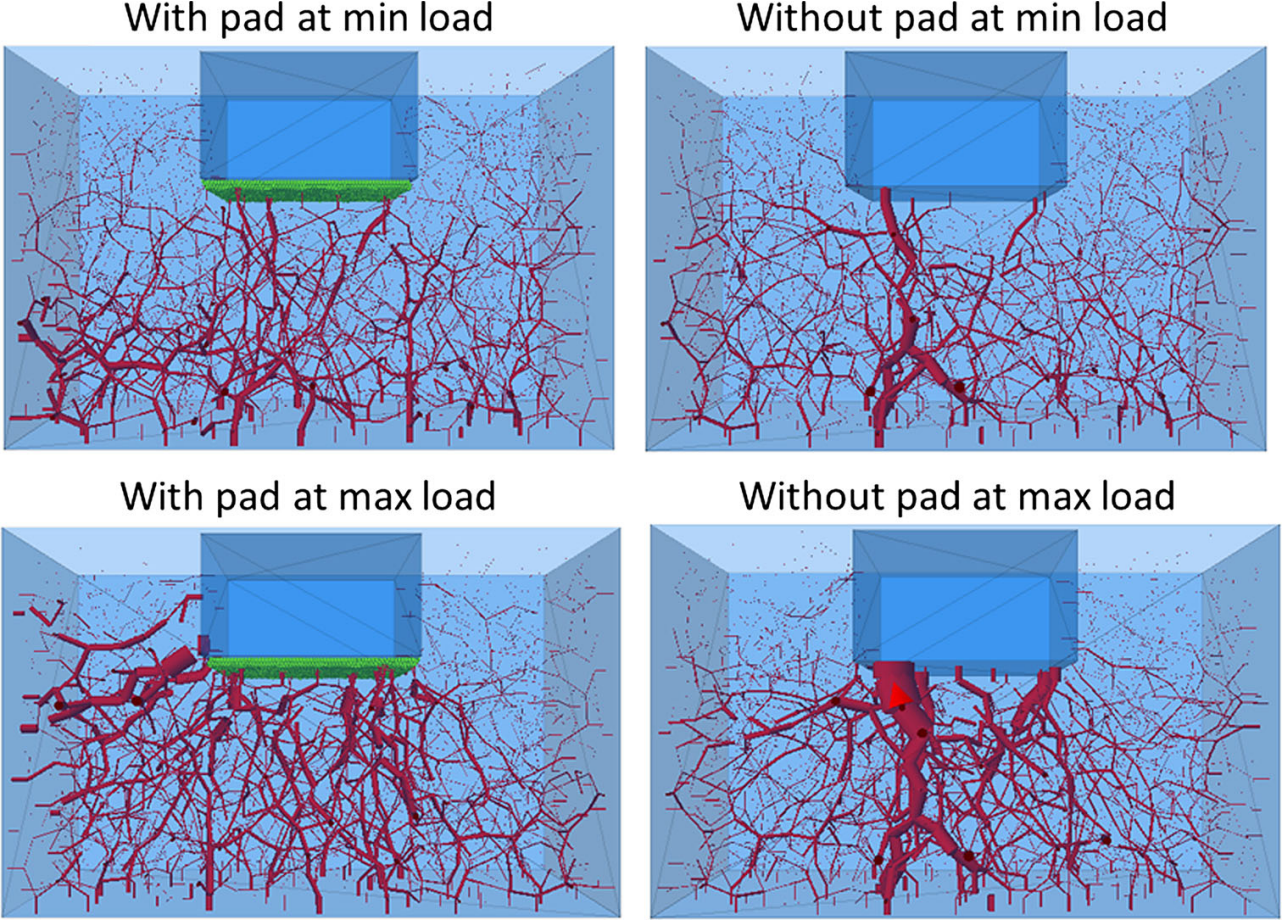
Contact force networks at 15th loading cycle

**Fig. 14:**
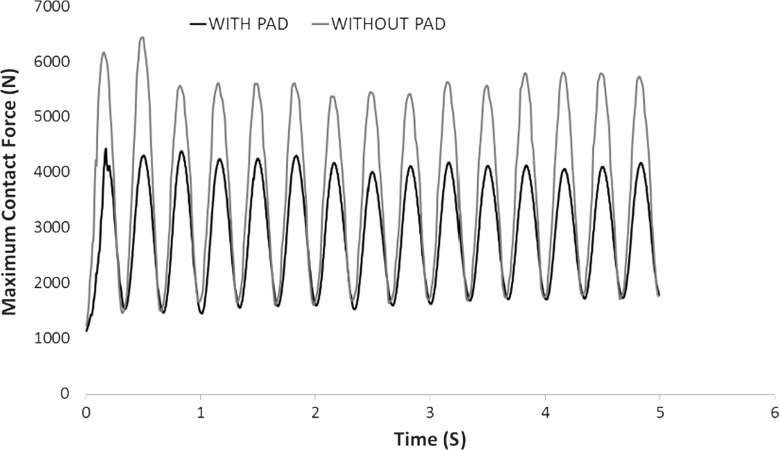
Comparison of max contact force

**Fig. 15:**
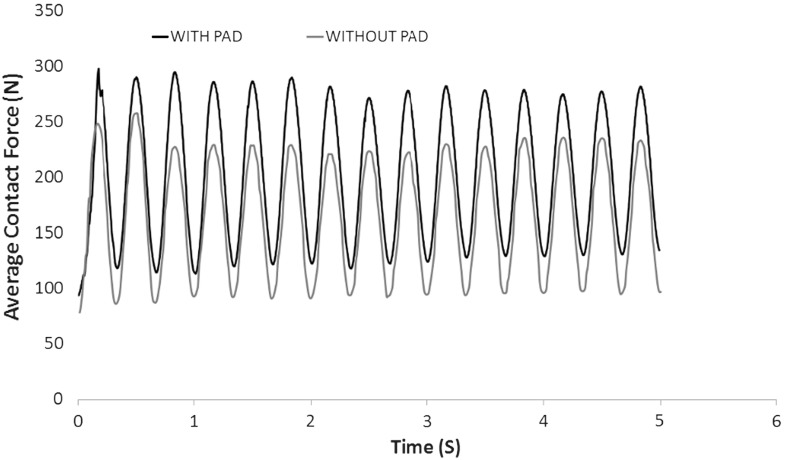
Comparison of average contact force

Figure 13 shows the contact force networks for both cases at minimum load and maximum load of the last loading cycle respectively. All lines of force are drawn to the same scale, where thicker lines represent larger forces. The comparisons indicate that the load distribution without a pad is mainly oriented vertically under the sleeper while the case with a pad seems to give greater diffusion of load laterally. Figures 14 and 15 compare the maximum and average contact forces respectively throughout the simulations. It is clear that the USP results in a smaller maximum contact force but a larger average contact force. This is to say, the application of the pad results in a more homogeneous load distribution and thus increases the number of ballast particles supporting the applied load.

**Fig. 16:**
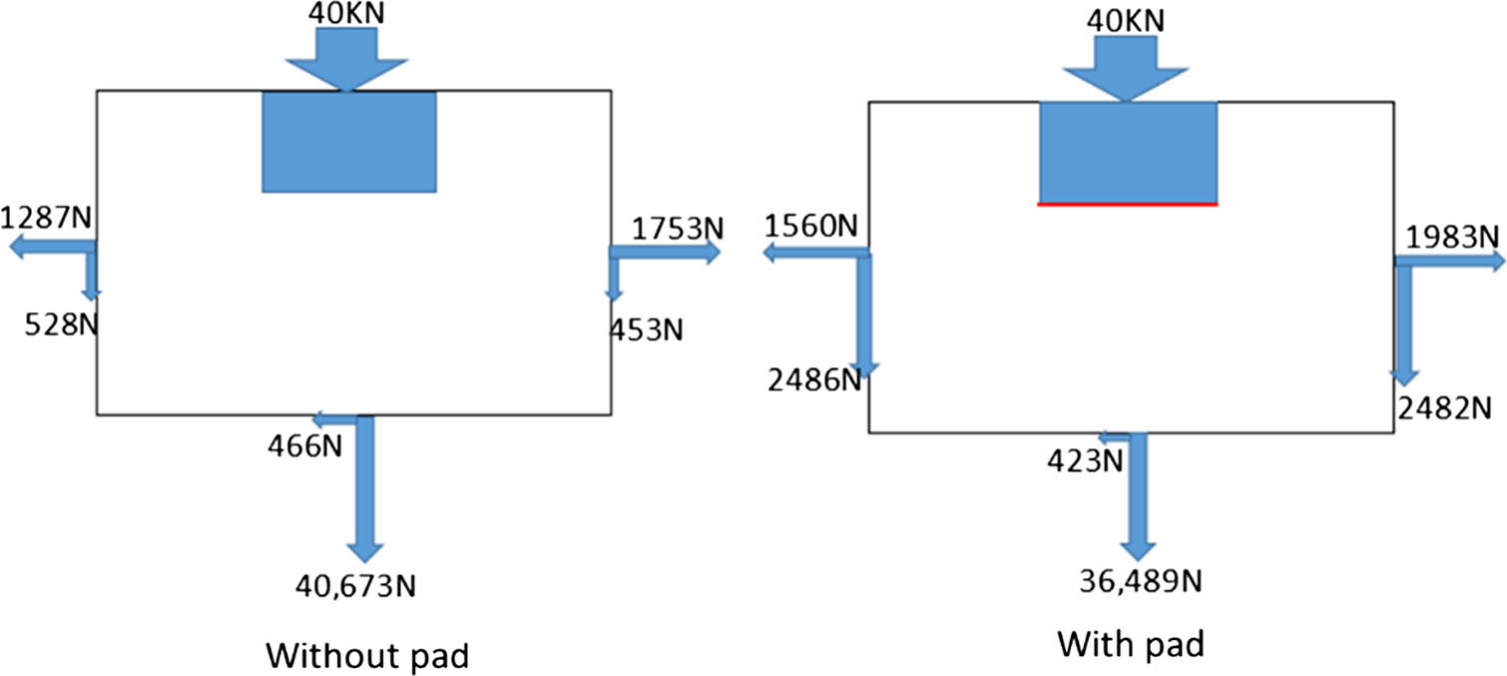
Comparison of the contact forces supported by boundary

As shown in Fig. 13, the application of USP seems to result in a greater diffusion of load laterally; the contact forces supported by the boundary have been summed to confirm this observation. Figure 16 shows an example of the contact forces supported by the side and bottom boundaries, calculated at the maximum load of the 15th cycle. It is noted that the weight of particles and the force acting on the front wall/back walls were not included to make the figure easier to read; these are the additional forces required to maintain equilibrium. For the case with pad, it is clear that the side boundary supports a larger contact force while the bottom boundary supports smaller contact force. Which is to say, the USP helps to transfer the load from sleeper to lateral direction whereas the loading force mainly concentrates vertically for the case without pad. Therefore, it is reasonable that the application of USP improves the track performance in terms of permanent settlement because there is more lateral diffusion of load.

Baghsorki et al [8] also tested another harder USP with stiffness of 0.228–0.331 $\hbox {N/mm}^{3}$ in their experiments. This pad was also modelled in this study; the parallel bond and contact bond properties were calibrated using the same method explained in previous section and their values are listed in Table 4.

**Fig. 17:**
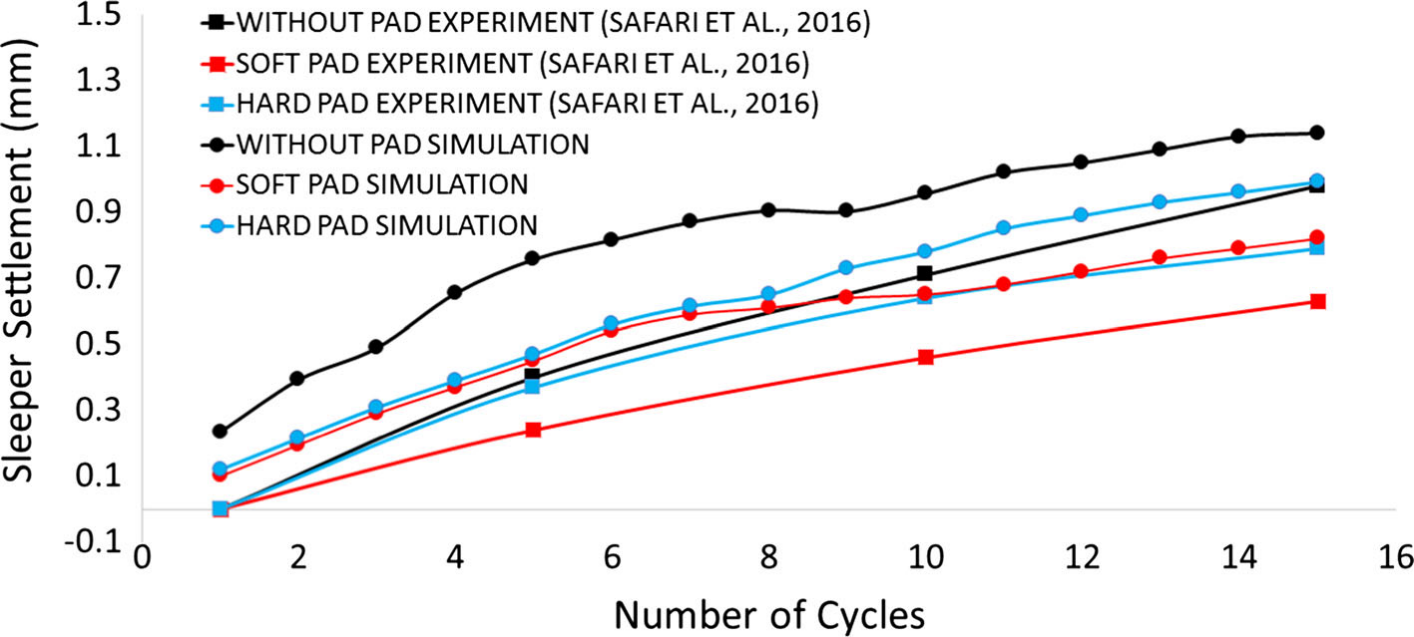
Comparison of sleeper settlement for various pads

**Fig. 18:**
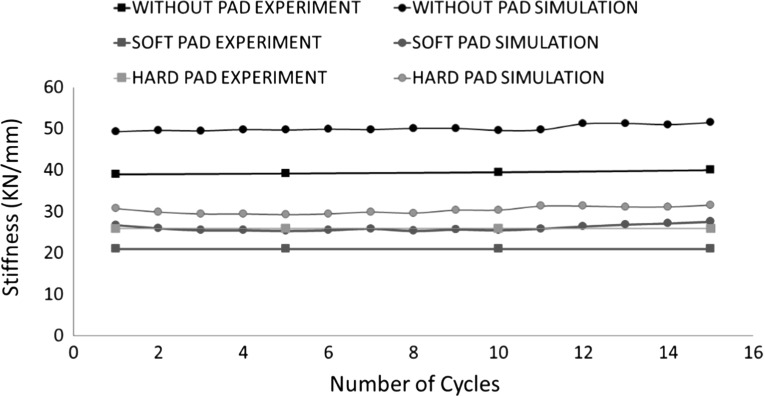
Comparison of trackbed stiffness for various pads

Figures 17 and 18 compare various pads with experimental results in terms of permanent settlement and trackbed stiffness respectively. Similar to the case using the soft pad, the DEM simulation gives a reasonable estimate for both settlement and stiffness. Moreover, the DEM simulation correctly predicts the effect of changing the pad stiffness found in experiments: a softer pad results in less permanent settlement and smaller trackbed stiffness.

## Particle abrasion

**Fig. 19:**
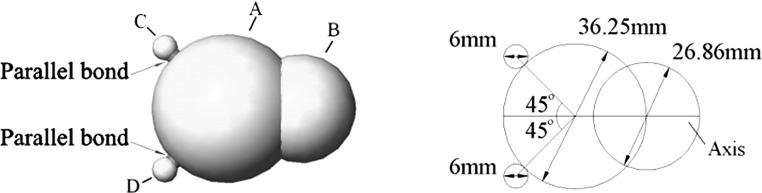
Breakable particle model

As particle breakage is one main causes of ballast degradation, investigating the effect of USP on ballast breakage is also an aim of this study. In terms of DEM modelling ballast breakage, the most challenging issue is to consider particle breakage and complex particle shape at the same time: e.g. Lobo-Guerrero and Vallejo [11, 12] published work on DEM of ballast degradation but only using circular particles and the same particle breakage model was employed to investigate the effects of pile shape and pile interaction on the crushing of granular material [33, 34]; it was found that the pile–particle interface plays an important role in governing both resistance force and particle degradation. The breakage model [11, 12] was also used to investigate the degradation of a granular base under a Flexible pavement [35], which has similar loading conditions to railway track and the crushing was found to initiate at the interface between the asphalt layer and the granular base and then continued to spread towards the bottom of the base layer. There are also a few studies where [36, 37] particle shapes were modelled but particle breakage was ignored. For limited studies considering both complex shape and breakage [31, 38–41], agglomerates of bonded balls was used to represent ballast particles, which makes the computation time-consuming and releases internal voids on fracture, causing artificial settlement. Considering most ballast degradation is not attributable to particle splitting but instead primarily particle abrasion [42, 43], Lu and McDowell [44] provided a breakable irregular shaped particle: a simple two-ball clump with two additional breakable asperities bonded to the clump surface (Fig. 19). Although this particle model cannot predict the track settlement quantitively [45], it successfully modelled the effect of ballast abrasion on permanent track settlement [37] and provided relatively fast computational speed. Considering the main aim of this section is to investigate the effect of using USP on particle abrasion, this particle model (Fig. 19) is used in this group of simulations. The particle and parallel bonds used in the simulation are those used by Lu and McDowell [44], and the parameters are listed in Table 5. The USP used in this simulation is the soft pad used in the previous section.

**Table 4:** The input bonds properties of hard pad

Parallel bond normal and shear stiffness	$2.76\,\times \,10^{8}$ Pa/m
Parallel bond normal and shear strength	$1\,\times \,10^{60}\,\hbox {Pa}$
Parallel bond radius multiplier	1
Contact bond normal and shear stiffness	$3\,\times \,10^{3}\,\hbox {N/m}$
Contact bond normal and shear strength	$1\,\times \,10^{60}\,\hbox {N}$
Normal and shear stiffness of mini sphere	$3\,\times \,10^{3}\,\hbox {N/m}$

**Fig. 20:**
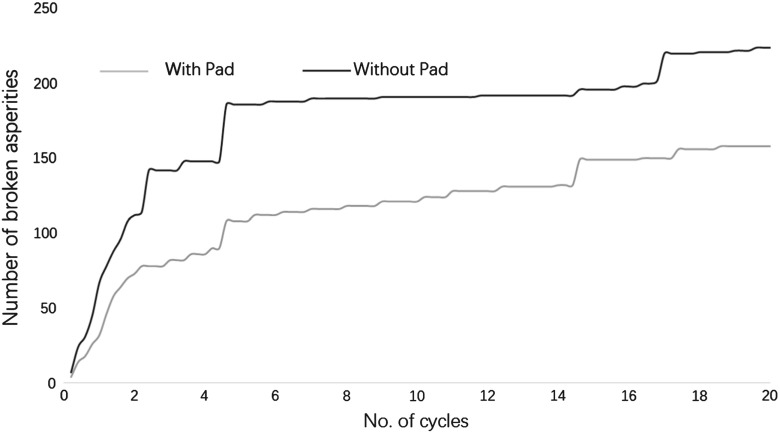
Comparison of broken asperities

**Fig. 21:**
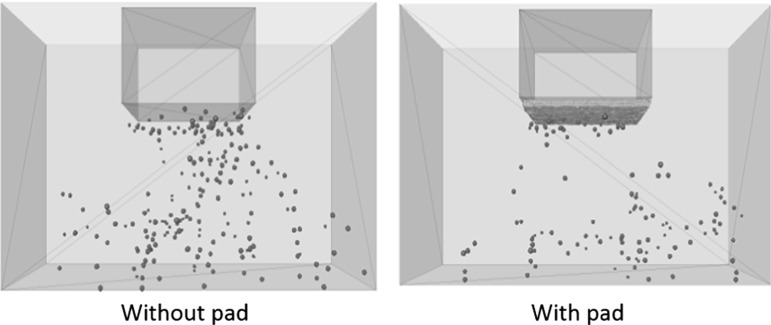
Comparison of the breakage locations

Figure 20 shows the total number of broken asperities during 20 loading cycles. It is clear that the USP reduces the number of broken asperities. The red spheres shown in Fig. 21 represent the locations of breakages that have occurred after 20 loading cycles. Compared with Figs. 12 and 13 which show the corresponding contact force networks, it is observed the breakages mainly occur at the sleeper/USP–particle interface and along the main contact force chains under the sleeper. It is clear that the number of breakages reduces for both of these zones of breakage when using an USP. For the particles in contact with the USP, the larger contact area reduces the contact pressure and therefore there are fewer particle abrasions. Regarding to the inter-particle breakage, Fig. 14 shows the maximum contact forces are generally smaller for the case with a USP, which explains why there are fewer inter-particle breakages.

**Table 5:** Input parameters of clump with breakable asperities

Input parameters of two-ball clump	
No. of clumps	1632
Density	$2960\,\hbox {kg/m}^{3}$
Damping coefficient	0.7 (default)
Friction	0.5
Poisson’s ratio	0.25
Shear modulus	28 GPa
Normal and shear stiffness of parallel bond	$3.5\,\times \,10^{12}\,\hbox {Pa/m}$
Normal and shear strength of parallel bond	$5\,\times \,10^{8}\,\hbox {Pa}$
Parallel bond radius multiplier	1

## Conclusions

DEM of a box test on ballast has been used to give micro mechanical insight into how Under Sleeper Pads improve the performance of ballasted railway track. This was modelled by representing the USP using three layers of small bonded spheres, and using a realistically shaped clump to represent the ballast particle. This DEM model cannot only qualitatively predict the improvement of applying a USP but also quantitively match the experimental results. The pads with different stiffnesses can also be simply modelled by changing the stiffness of bonds and spheres forming the pad. The micro-level analysis of DEM models indicates that by using a USP, more particles are effectively allowed to contact with the sleeper/pad composite system. It is also found that the contact force chains are concentrated underneath the sleeper for the case without USP whereas the USP provides a more homogeneous load distribution. Thus the USP transmits a smaller force vertically to the base, which is believed to be the reason why USP helps to reduce the track settlement. Particle abrasion was also considered by using a simple two ball clump with two breakable asperities, and it was found that breakage occurs mainly at the particle–sleeper interface and along the main contact force chains beneath the sleeper. Particle abrasion in both zones is reduced by the use of an under sleeper pad in the simulations. This work therefore lends credibility to the use of USPs in practice to reduce track settlement, and in the reduction of ballast abrasion caused by trafficking which ultimately causes a deterioration in track performance.
